# Hyperlipidemia and Obesity’s Role in Immune Dysregulation Underlying the Severity of COVID-19 Infection

**DOI:** 10.3390/clinpract11040085

**Published:** 2021-09-22

**Authors:** Christopher Khatchadourian, Christina Sisliyan, Kevin Nguyen, Nicole Poladian, Qi Tian, Faraaz Tamjidi, Bao Luong, Manpreet Singh, Jeremiah Robison, Vishwanath Venketaraman

**Affiliations:** 1 College of Osteopathic Medicine of the Pacific, Western University of Health Sciences, Pomona, CA 91766, USA; chris.khatchadourian@westernu.edu (C.K.); christina.sisliyan@westernu.edu (C.S.); kevin.nguyen2@westernu.edu (K.N.); nicole.poladian@westernu.edu (N.P.); qi.tian@westernu.edu (Q.T.); faraaz.tamjidi@westernu.edu (F.T.); bao.luong@westernu.edu (B.L.); 2 Department of Emergency Medicine, St. Barnabas Hospital Health System, Bronx, NY 10457, USA; preetysinghr1@gmail.com (M.S.); jer.b.robison@gmail.com (J.R.); 3 Department of Basic Medical Sciences, College of Osteopathic Medicine of the Pacific, Western University of Health Sciences, 309 E Second Street, Pomona, CA 91766, USA

**Keywords:** hyperlipidemia, SARS-CoV-2, COVID-19

## Abstract

Obesity and hyperlipidemia are known to be risk factors for various pathological disorders, including various forms of infectious respiratory disease, including the current Coronavirus outbreak termed Coronavirus Disease 19 (COVID-19). This review studies the effects of hyperlipidemia and obesity on enhancing the inflammatory response seen in COVID-19 and potential therapeutic pathways related to these processes. In order to better understand the underlying processes of cytokine and chemokine-induced inflammation, we must further investigate the immunomodulatory effects of agents such as Vitamin D and the reduced form of glutathione as adjunctive therapies for COVID-19 disease.

## 1. Introduction

The novel COVID-19 outbreak, caused by severe acute respiratory syndrome coronavirus 2 (SARS-CoV-2), as reported by the World Health Organization (WHO), has affected over 173 million individuals worldwide, resulting in almost 4 million deaths over the first 18 months of the outbreak [1]. The first case in this 2019 outbreak was originally identified in Wuhan, China, from where it quickly spread to trigger a worldwide pandemic [2].

SARS-CoV-2 is a part of the coronavirus family, which are RNA enveloped viruses known to be infectious towards mammals [3]. Symptoms of COVID-19 include, but are not limited to, fever, cough, shortness of breath, and other respiratory, cardiac, and muscular manifestations [4].

The detrimental effects of SARS-CoV-2 are secondary to the sudden and sustained inflammation that causes many of the symptoms present in COVID-19 infections [4]. The presence of pre-existing chronic inflammation can further increase the severity of symptoms of COVID-19 infections [5]. Such a phenomenon is thought to occur in obesity and hyperlipidemia, which have been shown to increase an individual’s susceptibility to disease states [6]. In a healthy individual, inflammation functions as a defensive response to maintaining one’s health; however, in these chronic pro-inflammatory states, this process is exacerbated and may lead to pathologic immune responses [7,8].

The World Health Organization reported in 2016 that 1.9 billion people worldwide were overweight and 650 million were obese. Considering that these values have tripled since 1975, and continue to rise, it is important to study and understand obesity-related conditions and complications [9]. Obesity is marked by an increase in adipose tissue which consequently results in the elevation of circulating free fatty acids functioning to activate the proinflammatory pathways (specifically, the NFκB pathway) to increase macrophage and cytokine concentrations [10]. Furthermore, the elevated low-density lipoprotein (LDL) particles seen in dyslipidemia are susceptible to oxidation (becoming Ox-LDLs), which further attract macrophages intending to uptake the ox-LDLs [11]. The presence and activation of macrophages, as seen in obesity, have been found to be extremely significant in the pathology of COVID-19 infection [12]. Previous research analyzing the mechanistic connections between COVID-19 infection and obesity has found obesity to be a risk factor in the severity of COVID-19 infection [6,13]. Macrophage production and increased release of cytokines contributes to an upregulation of the proinflammatory state seen in obesity [12].

Adipocytes play a vital role in the release of adipokines which may be either pro- or anti-inflammatory at local and systemic levels. In a healthy individual, these inflammatory states are in balance; however, obesity favors the release of proinflammatory agents, resulting in a baseline inflammatory state [5]. These marked increases in the levels of macrophage, cytokines, and pro-inflammatory adipokines maintained in a state of obesity result in a chronic inflammatory state [5,10,12]. This excessive upregulation of cytokine production, referred to as a “cytokine storm”, was often witnessed in severe cases of COVID-19 infection [12]. As the macrophage of individuals with obesity produce increased levels of pro-inflammatory cytokines, due to a chronic pro-inflammatory state, subsequent infection with COVID-19 will trigger the production of pro-inflammatory cytokines at higher levels than expected in individuals without chronic inflammatory disease [12].

This review focuses on studying immune pathways in order to propose mechanisms for potential therapies. We suggest the use of reduced glutathione and Vitamin D as effective therapeutics in decreasing the severity of disease manifestation based on their ability to reduce proinflammatory cytokines and increase anti-inflammatory cytokines, hence allowing for immune system homeostasis. 

## 2. Methods

**Figure 1:**
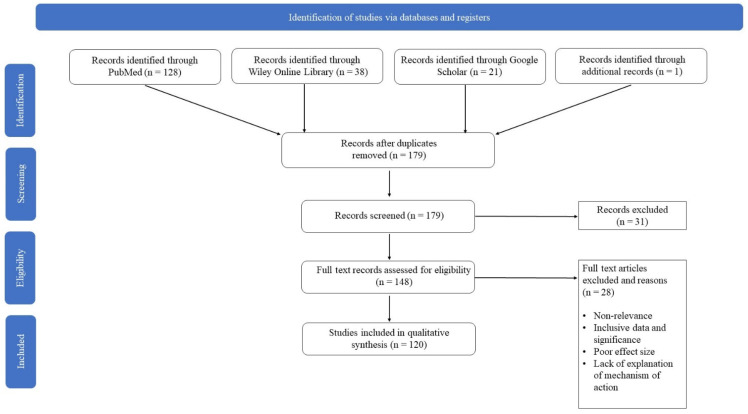
Chart outlining the article selection process.

This article is a comprehensive review exploring the impacts of obesity and hyperlipidemia on the pathogenesis of COVID-19 infections, as well as exploring potential therapeutic pathways. The literature for this review was obtained using the following search engines: PubMed, Wiley Online Library, and Google Scholar. Search terms common to each subsection of this review included any combination of the following: “COVID”, “Hyperlipidemia”, “SARS-CoV-2”, “COVID-19”, “dyslipidemia”, and “hypercholesterolemia”. Search terms specific to respective subsections included “Inflammation”, “Cytokine(s)”, “ACE2”, “Spike Protein”, “Furin”, “Apolipoprotein(s)”, “obesity”, “Nitric Oxide”, “Vitamin D”, “Dihydroxyvitamin D”, “Glutathione”, and “Reactive oxygen species”. Sources were selected based on the quality of methods and the quality, significance, and effect size of the results. Review articles were chosen based on the comprehensiveness of the topic of interest. Therapies were selected based on their proposed mechanism of action against inflammation as it related to the aforementioned inflammatory pathways. Further information regarding the article selection process is outlined in Figure 1.

## 3. Pathogenesis of COVID-19

**Figure 2:**
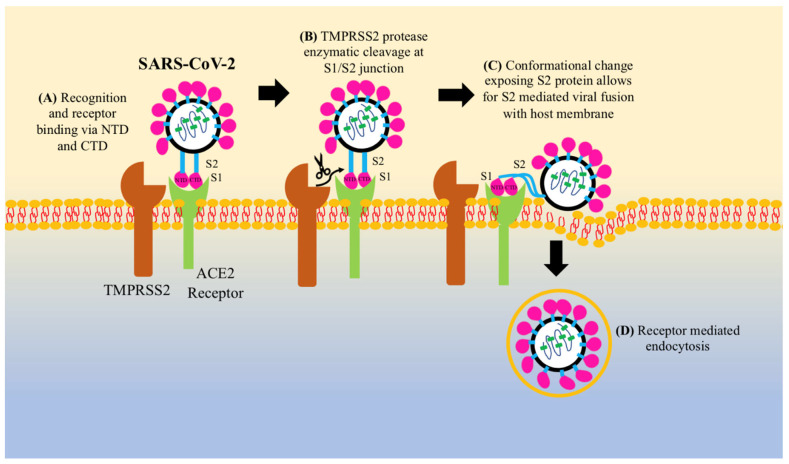
SARS-CoV-2 mechanism of entry into the host cell. SARS-CoV-2 is enveloped with a helical nucleocapsid. The envelope is coated by spike proteins, including an S1 and S2 subunit. (**A**) The S1 subunit has an N-terminal binding domain and a C-terminal binding domain which help with receptor binding and recognition. (**B**) Once S1 binds to an ACE2 receptor, it undergoes a conformational change with the help of the protease transmembrane protease serine 2 (TMPRSS2) to expose S2. (**C**) S2 serves as a viral fusion protein that integrates the viral membrane into the host cell membrane. (**D**) Once fused, SARS-CoV-2 can enter the cell via receptor mediated endocytosis.

While the complete pathogenic mechanism of SARS-CoV-2 is yet to be elucidated, the current literature has implicated some of the key features of this process. The SARS-CoV-2 virus is highly infectious and can be transmitted through respiratory droplets, aerosol, contact with contaminated surfaces, or fecal-oral transmission [14,15,16]. SARS-CoV-2 of the genus Betacoronavirus shares a similar genome and structure to other SARS-CoVs. These viruses are made up of 16 non-structural open reading frames (*ORFs*) *coding for the replicase complex*, and structural ORFs coding for *spike* (*S*), *envelope*, *membrane*, and *nucleocapsid proteins* [17]. The spike protein has two subunits: S1, which is composed of two domains, the N-terminal domain (NTD), which is involved in receptor recognition, and C-terminal domain (CTD) which binds to the target cell [18]; and S2, which contains viral fusion proteins to anchor the virus to the host cell membrane [19] (Figure 2). The SARS-CoV-2 S2 protein shares ~90% of its genomic profile with other SARS-CoVs [20]. Notable differences between SARS-CoV-2 and other SARS-CoVs exist among the S1 protein. Genomic analysis reveals two mutational variants: a Val483 insertion at the terminal end of the S-protein receptor binding domain and a unique Lys417 substitution in the receptor binding motif, both of which constitute more salt bridges and establish greater atomic interaction between SARS-CoV-2 and its receptor in the SARS-CoV-2 S1 protein region, corresponding to greater receptor binding affinity and greater stability [21]. This could explain the higher rate of infectivity compared to SARS-CoVs [20,22,23]. One way to measure infectivity is through evaluating the transmission rate of coronaviruses, which can be represented by the reproduction number (R_0_), a value that measures the average number of individuals that one infected individual can infect. The R_0_ for SARS-CoV-1 was 1.7–1.9 [24] and for Middle East Respiratory Syndrome (MERS) was <1 [24], while the R_0_ for SARS-CoV-2 ranges from 3.6–5.9 [25]. However, while more transmittable, SARS-CoV-2 shows lower severity and mortality rates of 2–4% as compared to SARS-CoV-1 (10–11%) and MERS (34–37%) [26].

SARS-CoV-2, like other Betacoronaviruses, has affinity for three receptor binding domains (RBDs) on target cells, the most important of which is the angiotensin-converting enzyme 2 (ACE2) receptor, as it is required for the SARS-CoV-2 cell entry process [27]. SARS-CoV-2 responsive ACE2 receptors have been identified mainly throughout the lung epithelial cells, type I and II pneumocytes, and club cells, as well as within nasal goblet secretory cells and ileal absorptive enterocytes, hence explaining the presence of upper respiratory tract symptoms as well as gastrointestinal disturbances [28]. Unique to SARS-CoV-2 is the S1/S2 junction site that contains a pre-cleavage site made up of multiple arginine residues, which is targeted by furin [29]. Furin is a protease, also known as PCSK3, which is ubiquitously expressed throughout cells and functions in cleavage of only certain motif sequences [30]. Furin pre-cleavage affects pathogenicity as it induces viral fusion to the cell membrane [31]. Furin prepares the S protein for further cleavage at the S1/S2 junction which occurs via two proteases, cell surface transmembrane protease serine 2 (TMPRSS2) or cathepsin-L, which are proteases expressed in endosomal compartments that activates the S protein when it is taken up by endocytosis [32]. Cleavage of the S protein allows for membrane-to-membrane fusion for viral entry [33]. Recent data demonstrate a preferential utilization of cell surface TMPRSS2 activation of the S protein independent of endosomal cathepsin [34,35]. 

Viral endocytosis occurs either by direct plasma membrane attachment, creating a fusion pore for viral RNA transfer, or clathrin-mediated invagination of the plasma membrane to form intracellular vesicles [36,37]. Once the viral RNA is released into the cell, it uses host cell machinery for viral replication and assembly. SARS-CoV-2’s entry into the cell alters the lipid profile. Lipids are required for proper metabolism as they play an essential role in cellular signaling, structural integrity, energy storage, and transport. Lipid dysregulation (in the form of obesity and hyperlipidemia), however, has negative consequences on cellular functioning by allowing for easier viral entry into host cells, leading to viral infections [38].

The severity of SARS-CoV-2 is typically attributed to the cytokine storm, which can be induced by lipid accumulation and oxidation. Analysis of the lipidemic profile of SARS-CoV-2-infected patients reveals an accumulation of lipid droplets such as diacylglycerols, triacyclglycerols, glycerophospholipids, cholesterol, and ceramides, promoting viral assembly [39,40]. In particular, higher cholesterol levels were associated with greater levels of SARS-CoV-2 infectivity via enhanced viral entry due to increased viral fusion to the host membrane [38]. There is also a marked elevation of lipogenic markers such as sterol regulating element-binding protein (SREBP), a transcription factor for cholesterol synthesis; peroxisome proliferator-activator receptor gamma (PPARγ), a transcription factor involved in lipid uptake and adipogenesis; and CD36, a membrane protein that aids in long chain fatty acid uptake [41].

Accumulated lipids, particularly LDLs, are subject to oxidation, and oxidized LDLs lead to several downstream consequences such as a cytokine storm. COVID-19’s severity and mortality are mainly attributed to this immune process, a hyperinflammatory state which contributes to systemic infection, organ failure, and a diminished immune response. Thus, further investigation of the lipid dysregulation seen in COVID-19 may provide important insight for therapeutic avenues to regulate cytokine dysfunction. A particularly interesting relationship is that of obesity and SARS-CoV-2. Obesity is characterized as a low-grade chronic inflammatory state that utilizes a similar molecular pathway as SARS-CoV-2 [42]. The elevated levels of adipose cells in obesity can lead to increased levels of adipokines, such as leptin. Leptin accumulation can promote leptin and insulin resistance, leading to hyperglycemia [43], and SARS-CoV-2 may further contribute to hyperglycemia, though this mechanism is less clear and needs further exploration [42,44]. Hyperglycemia and lipid accumulation induce lipid oxidation through advanced glycation end products, which can set off a massive release of pro-inflammatory cytokines, leading to the cytokine storm [45]. In addition, obesity can lead to arterial hypertension characterized by increased ACE2 receptor expression which SARS-CoV-2 can use for cell entry [42]. The virus can utilize these pre-existing non-homeostatic lipid levels to mediate enhanced entry, replication, assembly, and budding, resulting in greater infectivity. A clinical study of hospitalized patients with SARS-CoV-2 showed a significant association between patients with BMI >24 and disease exacerbation, pneumonia, and ICU admission [46]. The concurrent inflammatory and molecular processes occurring in patients with comorbid conditions, such as obesity, which alter lipid profiles, can be exploited by SARS-CoV-2 to cause exacerbated disease, leading to higher rates of hospitalization and fatality when compared to their healthier counterparts [46,47,48]. 

## 4. Cholesterol’s Role in COVID-19 Pathogenesis

In the liver, cholesterol is packaged with Apolipoprotein B (Apo-B), Apolipoprotein E (Apo-E), and several other substrates to form very low-density lipoprotein (VLDL). VLDL is then metabolized to form low-density lipoprotein (LDL), which transports cholesterol to peripheral tissues and ultimately to the liver, where it is removed from circulation. Uptake of LDL in both peripheral tissues and the liver takes place via Apo-E recognition by the LDL receptor on cellular membranes [49].

**Figure 3:**
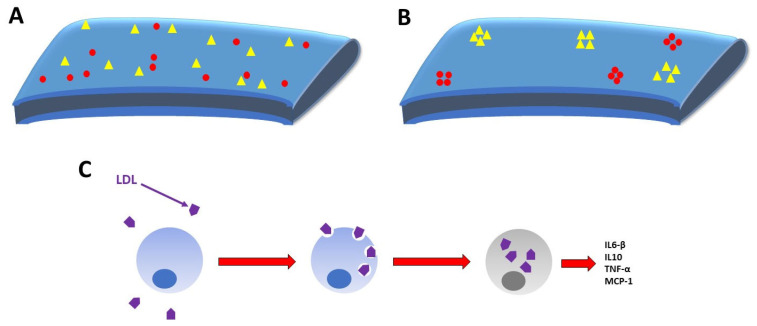
Epithelial cell surface membrane with (**A**) diffusely assorted ACE2 receptors (red circles) and furin (yellow triangles) and (**B**) increased furin and ACE2 receptor aggregation onto lipid rafts following Apo-E induced internalization of cholesterol. (**C**) Macrophages (blue) uptake oxidized LDL (purple), thus becoming foam cells (gray) which produce greater levels of circulating cytokines.

Obesity and age are significantly associated with increased cholesterol levels in lung tissues in mammalian animal models. Elevated LDL cholesterol can increase SARS-CoV-2 infectivity and internalization into epithelial cells. Cholesterol internalized into epithelial cells via Apo-E significantly increased the aggregation of furin and ACE2 receptors onto focal areas of the cell membrane known as lipid rafts (Figure 3). Aggregation of ACE2 receptor and furin onto lipid rafts is significantly associated with increased endocytosis and infectivity of the SARS-CoV-2 virus. Furthermore, when cholesterol was experimentally extracted from cell membranes, entry of more than 90% of SARS-CoV-2 mimic particles was inhibited [50].

The aforementioned SARS-CoV-2-associated cytokine storm involves the overproduction of cytokines which include but are not limited to Interleukins 1 (IL-1), 6 (IL-6), and 10 (IL-10), tumor necrosis factor alpha (TNF-α), and monocyte chemoattractant protein 1 (MCP1) [51,52,53]. LDL, by exposure to endothelial oxidative agents such as enzymes, metal ions, and Peroxynitrous acid (a product of nitric oxide and superoxide anion reactions), can undergo oxidation [54]. Oxidized LDL (Ox-LDL) is associated with increased circulating levels of TNF-α, a pertinent component of the cytokine storm [55]. Ox-LDL is promptly taken up by macrophages which transition into foam cells upon intracellular accumulation of the lipoprotein (Figure 3). This intracellular cholesterol enhances expression of TNF-α and IL-6-β in foam cells (Figure 3) [56]. LDL also induces the release of IL-1, IL-6, IL-10, and MCP1 in cultured monocytes (Figure 3) [57]. Increased production of these SARS-CoV-2-relevant cytokines are associated with greater levels of LDL and could potentially render one more susceptible to the deadly cytokine storm seen in SARS-CoV-2 infection.

High-density lipoprotein (HDL), like LDL, is a cholesterol transporting lipoprotein. Unlike LDL, HDL is involved in the reverse transport of cholesterol from peripheral tissues back towards the liver [58]. According to a cross-sectional study by Masana et al., lower levels of high-density lipoprotein (HDL) cholesterol are associated with a poorer COVID-19 prognosis [59]. In an observational study, Wang et al. stated that HDL cholesterol levels were lower in COVID-19-infected adults (0.78 vs. 1.37 mmol/L in age- and gender-matched healthy controls) and that this was associated with a greater probability of developing severe disease [60]. Disease severity could be impacted by HDL’s effects on the production of inflammatory cytokines. HDL induces a transcriptional repressor known as activating transcription factor 3 (ATF3), which limits the production of the toll-like receptor (TLR)-induced cytokines IL-6, IL-12, and TNF [61]. HDL also promotes the efflux of cholesterol from foam cells, effectively preventing their formation. Furthermore, ApoA-I, the principal structural protein of HDL [56] can remove oxidized cholesteryl esters from LDL, thus reducing ox-LDL levels and minimizing the formation of inflammatory foam cells [62].

## 5. Obesity and SARS-CoV-2 Infection

Currently, the world is facing two pandemics—obesity and COVID-19. Obesity results from genetic, behavioral, and environmental factors that derive from an imbalance between energy intake and expenditure [63]. The worldwide prevalence of obesity has nearly tripled from 1975 to 2016 [64], contributing to the progression of cardiovascular diseases, diabetes, respiratory diseases, venous thromboembolism, and certain cancers such as endometrial and esophageal adenocarcinomas [65,66,67,68,69].

The physiologic structure and function of adipose tissue changes with obesity, leading to both hyperplasia and hypertrophy. As the adipocytes expand, there is insufficient vascularization, leading to hypoxia, necrosis, and ultimately an inflammatory response [5,70,71,72]. As a result, hypertrophic adipose tissues, particularly visceral adipose tissues, develop a chronic low-grade inflammatory environment rich in leukocytes, such as macrophages, T cells, B cells, dendritic cells, natural killer cells, and mast cells [70,73,74,75]. High fat diet-induced obesity increases the ratio of M1 pro-inflammatory macrophages to M2 anti-inflammatory macrophages (M1:M2). M1, which are classically activated macrophages, are induced by IFN-γ from Th1 cells and function to release pro-inflammatory cytokines such as IL-6, IL-1β, inducible nitric oxide synthase NOS (iNOS), and TNF-α [76,77,78]. Expanded adipose tissues also increase the activation of caspase-1, which yields more nod-like receptor family pyrin domain containing-3 (NLRP3) inflammasomes that regulate and increase pro-inflammatory cytokines such as IL-1β and IL-18 [79,80]. Furthermore, the upregulation of pro-inflammatory cytokines, such as TNF-α regulated by M1 macrophages, act on adipocytes and activate IκB kinase-β (IKK-β) and mitogen-activated protein kinase (MAPK), which inhibit the phosphorylation on insulin receptor substrates 1 and 2, inducing an insulin-resistant state that ultimately amplifies inflammation [81]. The defect in insulin signaling is further amplified by upregulation of iNOS, induced by inflammatory cytokines in adipose tissues [82]. More significant amounts of iNOS yield more reactive nitrogen and oxygen species, which ultimately augments the levels of oxidative stress, thereby contributing to various pathological conditions such as insulin resistance and diabetes, cardiovascular diseases, and respiratory complications [83,84,85]. As a result, obesity-induced changes further contribute to the upregulation of pro-inflammatory cytokine secretion during the cytokine storm seen in COVID-19 [86].

In COVID-19 infections, a hyperactive immune response marked by cytokine overproduction leads to acute respiratory disease syndrome (ARDS), the pathogenesis of which involves inflammatory destruction of the alveolocapillary membrane, leading to increased lung permeability and pulmonary edema, which clinically can advance to hypoxia and respiratory failure [87]. Furthermore, when the respiratory system is invaded by a pathogen, there are subsequent immune responses leading to increased cytokine production in the lungs specifically (TNF-α, IL-1β, and IL-6) and lipopolysaccharides (LPS). Obesity exacerbates underlying pulmonary disorders as the vascular system fails to adequately perfuse the heightened number of enlarged adipocytes, resulting in hypoxia and further inflammation and apoptosis [88,89]. In obesity, the cytokine surge causes endothelial dysfunction (an alteration in the balance between vasodilatory and vasoconstricting agents in the vascular endothelium), leading to lung endothelial cell apoptosis and the formation of atherosclerotic vascular plaques [90,91,92]. Additionally, ACE2 expression is upregulated in hypertrophic adipose tissue, allowing for the vascular endothelium of obese patients to be targeted by the SARS-CoV-2 virus at a higher rate since the ACE2 gene is a receptor for the virus [71,93,94]. Post-mortem histology in one study shows evidence of direct SARS-CoV-2 viral infection on endothelial cells and endothelial inflammation that led to multiple organ failure and ultimately death [95]. Thus, the increase in adipose tissue from obesity can cause an upregulation of pro-inflammatory cytokines, disrupting the immune response and amplifying the systemic inflammation seen in SARS-CoV-2 infected patients, resulting in the development of severe organ failures and disease outcomes.

## 6. Vitamin D and COVID-19

Vitamin D is a fat-soluble protein that plays a major role in bone mineral density and calcium homeostasis [96,97]. It initially exists as an inactive compound, and is subsequently hydroxylated in the liver, followed by further conversion to the active form 1,25-dihydroxyvitamin D in the kidneys [98]. Studies have shown that the active form of Vitamin D plays a regulatory role in signaling pathways that involve immune responses and inflammation [97]. A study by Miroliaee and Tram showed that Vitamin D (300,000 units) administration decreased IL-6 levels in ventilator-associated pneumonia patients [99]. Vitamin D inhibits monocyte production of inflammatory cytokines such as IL-6 through inducing MAPK Phophatase-1 (MKP-1) expression. MKP-1 switches off p38 signaling and cytokine production in monocytes after an inflammatory stimulus, thereby reducing cytokine storm [100]. Among the elevated cytokines that are discovered in severe COVID-19 patients, the blood level of IL-6 was particularly elevated [52,101,102]. IL-6 is a pleiotropic cytokine that regulates immune response and cell metabolic and regenerative processes [103]. IL-6 can induce either an anti-inflammatory effect or a pro-inflammatory effect based on the receptors that it binds to. An anti-inflammatory effect is induced when IL-6 is bound to a transmembrane receptor via classic signaling. A pro-inflammatory effect is induced when it binds to a soluble receptor that leads to the recruitment of mononuclear cells, which contributes to COVID-19 cytokine storm [104]. Studies show that Vitamin D can effectively lower IL-6 and other inflammatory biomarker levels, which reduces the pro-inflammatory effect. In various respiratory diseases such as influenza, COPD, and upper respiratory tract infections (URTI), Vitamin D has also been proven to be effective in preventing infections and reducing the number of acute exacerbations [105,106,107]. The presence of Vitamin D receptors (VDR) on immune cells and the discovery of local hydroxylase activity that converts inactive form of Vitamin D to its active form leads to the hypothesis that Vitamin D has a direct effect on immune cells [106]. Therefore, it is postulated that Vitamin D can enhance innate defense mechanisms against viral pathogens while inhibiting pulmonary inflammatory responses [107]. Even though there is no direct clinical data that show Vitamin D alone can effectively treat respiratory viral infection such as COVID-19, there are multiple studies showing a relationship between vitamin D and inflammatory markers such as IL-6 and TNF-alpha. In animal and in vitro cell models, vitamin D has been shown to downregulate these pro-inflammatory cytokines. In addition, vitamin D levels have been shown to be lower in patients hospitalized with severe COVID-19 infections. While changes in vitamin D levels have not proven a causal relationship, these studies suggest a potential mechanism for the therapeutic benefit of vitamin D [108,109]. 

## 7. GSH and COVID-19

Glutathione (GSH) is an intracellular tripeptide found in all living cells, essential for mitigating oxidative stress. Glutathione peroxidase 4 (GPx4) is an antioxidant responsible for facilitating detoxification via the conversion of reactive oxygen species (ROS) to water by oxidizing GSH. As the pathogenesis of SAR-CoV-2 becomes known, the role of the two most common co-morbidities, obesity and hyperlipidemia, becomes a cause of concern due to their underlying inflammation, with both leading to a baseline increase in pro -inflammatory cytokines. Obese individuals are also noted to have higher Ferritin levels [110]. The relationship between ferritin and cytokine release was demonstrated in a study by Tran et al., where injecting rats with pro-inflammatory cytokines lead to increased Ferritin levels [111].

It has been shown that ferritin and IL-6 are useful in monitoring disease progression and severity of cytokine storm in COVID-19 patients; recovering patients were observed to have down-trending ferritin and IL-6 levels [112]. In addition to the ROS generated by iNOS, ROS are also generated by NADPH oxidase 4 (NOX4), which is up-regulated following viral infection in the lungs [113]. Furthermore, an increase in ferritin leads to additional formation of ROS, and the accumulation of ROS increases lipid peroxidation, which then induces ferroptosis that can cause tissue damage in the lungs [114]. Thus, SAR-CoV-2 infection coupled with obesity can increase the probability of patients progressing to the final stage of inflammation and leads to development of ARDS. Because ROS initiates lipid peroxidation, SAR-CoV-2 infection can increase oxidation of LDL. By removing the initiators, GPx can decrease the rate of LDL oxidation. The presence of GPx activity is noted in both LDL and HDL. The amount of baseline diene conjugation serves as an indicator of oxidized LDL in vivo [115]. Dose-dependent liposomal GSH resulted in prolongation of the lag time required for initiation of conjugated-dienes formation when incubated with LDL and HDL [116]. GSH has been shown to increase the levels of HDL, a promoter of the efflux of cholesterol from macrophages, which plays a role in the availability of ACE2 receptors and interferes with the early infection phase of SARS-CoV-2. Through the discussed mechanisms, we propose that GSH adjunct therapy can decrease the inflammatory injury of the lungs by decreasing ROS, IL-6, and Ox-LDL. Therefore, GSH may be effective in promoting better airway pressure and decreasing the severity of SAR-CoV-2 infection. 

The role of GSH in promoting immune system homeostasis by decreasing pro-inflammatory cytokines, notably IL-6, has been shown in diseases such as Human Immunodeficiency Virus and diabetes [117]. These studies showed that the patients had low GSH levels and high IL-6, which normalized with GSH supplementation. It has been demonstrated that SAR-CoV-2 patients with moderate and severe illness had lower levels of GSH, higher ROS levels, and greater redox status (ROS/GSH ratio) as compared to patients with mild symptoms [118]. A clinical trial (NCT04570254) showed that N-acetyl-cysteine (NAC), a precursor of GSH, in moderate doses could decrease inflammation and improve prognosis in SAR-CoV-2 infection [119]. A trial of PO or IV GSH was used in two patients with an improvement in dyspnea observed within 1 h of use. Continuous use of higher doses was effective in further relieving respiratory symptoms [120]. Currently, there are five ongoing clinical trials exploring the efficacy of GSH as well as GSH precursors in preventing lethal development of SAR-CoV-2 infection. 

## 8. Conclusions

During this pandemic, many physicians may have an added clinical challenge of treating patients with multiple disease states during a SARS-CoV-2 infection. With several vaccine candidates emerging, there is hope that mortality and disease burden is significantly reduced for vulnerable populations with pre-existing comorbidities. Infections pose a greater risk for patients with diabetes, obesity, and high cholesterol as these conditions have baseline inflammation. Here, we presented current literature to support that preexisting metabolic conditions can exacerbate the cytokine storm induced by SARS-CoV-2. Furthermore, we hypothesized that Vitamin D and GSH, both of which have been shown to modulate the immune system to homeostasis in other inflammatory states, may temper the disproportional immune response in COVID-19 patients. While scientific advances are rapidly on the rise, thus far, only about 11% of the global population has been fully vaccinated. We hope that these potential adjunctive therapeutics may help decrease the severe morbidity of COVID-19. This may be beneficial as these are vitamins and antioxidants which are readily available, as the current treatments, remdesivir and corticosteroids, along with vaccination may not be as immediately available to all individuals across the globe.

## Data Availability

See references section.
